# A challenging case of Down syndrome regression disorder

**DOI:** 10.1055/s-0046-1819661

**Published:** 2026-05-12

**Authors:** Letícia Klabinske Marques Monteiro, Aimê de Paula Santos, Helena Santos de Moura Lima, Rejane de Souza Macedo Campos, Jesus Manoel Bernardez Gandara, Luziany Carvalho Araújo, Breno José Alencar Pires Barbosa

**Affiliations:** 1 Universidade Federal de Pernambuco, Empresa Brasileira de Serviços Hospitalares, Hospital das Clínicas, Serviço de Neurologia, Recife PE, Brazil.; 2 Universidade Federal de Pernambuco, Centro de Ciências Médicas, Recife PE, Brazil.; 3 Hospital Israelita Albert Einstein, São Paulo SP, Brazil.; 4 Real Hospital Português de Beneficência em Pernambuco, Recife PE, Brazil.; 5 Universidade Federal de Pernambuco, Empresa Brasileira de Serviços Hospitalares, Hospital das Clínicas, Serviço de Radiologia, Recife PE, Brazil.

**Keywords:** Iron Metabolism Disorders, Intellectual Disability, Cognition

## Abstract

Down syndrome regression disorder (DSRD) is a rare cause of neuropsychiatric regression observed in previously-healthy individuals with Down syndrome (DS). There have been reports of brain iron accumulation in the basal ganglia of patients with DS and DSRD, although the underlying etiology remains unclear. The current study aims to report, through detailed neuroimaging, laboratory tests, and clinical data findings, the case of a patient with DS who experienced a significant loss of abilities, accompanied by neuroimaging findings indicative of abnormal brain iron accumulation. While an extensive investigation with blood, cerebrospinal fluid (CSF), and genetic markers was unremarkable, magnetic resonance imaging (MRI) scans revealed abnormal iron deposition and calcifications in the globus pallidus. The abnormal iron accumulation in the basal ganglia of patients with DSRD could be a potential neuroimaging marker for this condition.

## CLINICAL VIGNETTE

A 32-year-old male patient with Down syndrome (DS) presented with episodes of syncope and progressive motor decline throughout the past 5 years. Initially, he showed reduced interaction, increased apathy, and cognitive slowing, developing a reliance on communication devices to type his responses. The family reports that, during the same period, episodes of pallor and fainting in an orthostatic position occurred, lasting up to 60 seconds, with rapid recovery of consciousness. There was no report of limb jerks, tongue biting, or sphincter release. These events were initially rare, but became more frequent throughout the following months, and they were attributed to orthostatic hypotension after appropriate investigation, which will be further detailed herein. Other autonomic symptoms included urinary urgency, mild-to-moderate intestinal constipation, and–more recently–sleep behaviors that resembled vivid nightmares. In addition, he developed insidious, progressive global motor slowing and required assistance with standing and walking at some point, becoming dependent on all activities of daily living after 4 to 5 years of the first symptoms. In the past year, he showed irritability towards his family, but other symptoms, such as psychosis or compulsive behaviors, were absent. His parents were unaware of a similar family history.

During the clinical examination, he was alert, in good general condition, well-colored and hydrated, with stable vital signs. The cognitive assessment was quite limited, due to significant impairment in cognitive processing. He had severe difficulty verbalizing spontaneously and struggled to name the figures from the National Institutes of Health Stroke Scale, remaining almost mute during the evaluation. He responded to simple commands and yes-or-no questions with a delay of up to 45 seconds. During the physical examination, generalized bradykinesia, mild, slightly asymmetrical hand rigidity (more pronounced on the right), and a stooped posture were observed. No involuntary movements, such as tremors, dystonia, myoclonus, or stereotypies were observed. There were no pyramidal signs, nor asymmetries in the superficial or deep reflexes. The sensitive and cerebellar tests were affected by extreme cognitive and movement slowness, but there was no evident decomposition, dysmetria, nor areas of hypoesthesia. On the cranial nerve examination, facial hypomimia and hypophonia were evident. Despite limited verbal interaction, he made appropriate eye contact and exhibited impaired pursuit eye movements. The patient was wheelchair-bound and had great difficulty maintaining an upright position, requiring support.

A comprehensive evaluation involving various diagnostic modalities and the opinions of specialists in neurology and genetics was conducted. The progressive corticosubcortical pattern of involvement, along with significant dysautonomia, led to the hypothesis of neurodegenerative diseases, particularly atypical Parkinsonian syndromes such as multiple system atrophy. Other possibilities at this point included inborn errors of metabolism, autoimmune encephalitis, early-onset Alzheimer's disease, and DS regression disorder (DSRD). A thorough blood workup, including hormonal, autoimmune, metabolic, infectious, and cardiovascular studies, was unremarkable, except for nonspecific, mildly elevated antinuclear antibody and anti-thyroid peroxidase levels, as well as pleocytosis in the cerebrospinal fluid (CSF) ( Table 1 ).

**Table 1:** Investigation conducted with laboratory, neuroimaging, electrophysiological, and cerebrospinal fluid studies

*Laboratory and imaging data*		*Results*
Routine		Full blood count and metabolic panel: normal; Urinalysis: normal; Homocysteine: 6.51 µmol/L; total iron: 82 µg/dL; ferritin: 115.01 ng/mL; vitamin B12: 412 pg/mL; vitamin D: 44.3ng/mL
Cerebrospinal fluid		(2024) glucose: 55 mg/dL; protein: 37 mg/dL; cell count: 20 (80% lymphocytes, 20% monocytes); VDRL: NR
		(2020) glucose: 76 mg/dL; protein: 20 mg/dL; cell count: 1
		(2018) glucose: 67 mg/dL; protein: 20 mg/dL; cell count: 1; gamma-globulin: 1.11% of the total protein
Inflammatory and autoimmune	May 2024	Anti-thyroid peroxidase: 350 UI/mL; antithyroglobulin antibody: NR; thyroid-stimulating hormone: 1.53 mUI/L; free thyroxine: 1.05 ng/dL; thyroglobulin: 60 ng/mL; antinuclear antibody: 1/80 (homogenous pattern); CRP: 8.51 mg/dL; total complement activity (CH50, total complement assay): 44.8 U/mL; C4: 20mg/dL; IgA, IgG, and IgM: normal
	October 2023	Anti-Ro, anti-La, anti-Sm, Anti-dsDNA, anti-double-stranded Deoxyribonucleic acid, ANA, and RF: NR
	March 2023	CSF antibody panel: negative for onconeural and neural surface antibodies
Infectious	Negative	HIV, syphilis, and hepatitis
Exome		Trisomy 21, a likely-pathogenic heterozygous variant in the *ACADM* gene, and a likely-pathogenic heterozygous variant in the *MEGF10* gene
Electrophysiological studies	Negative	EMG of the 4 limbs,* evoked potential test (2024); EEG (2022; 2016)
Neuroimaging studies	Negative	TCD,** lumbar spine MRI, and cervical spine MRI (2024); skull and sella MRI (2020); and brain 18F-FDG PET (2019)
	Altered	Skull MRI (2022): basal ganglia hypointensities on SWI; skull CT scan (2016): small and symmetrical calcifications on the basal ganglia
Cardiovascular studies	Negative	ECG, Holter ECG monitoring

Abbreviations: 18F-FDG, [18F]Fluorodeoxyglucose; *ACADM* , *acyl-CoA dehydrogenase medium chain* ; ANA, antinuclear antibody; Anti-dsDNA, anti-double-stranded Deoxyribonucleic acid; C4, complement component 4; CH50, total complement assay; CRP, C-reactive protein; CSF cerebrospinal fluid; CT, computed tomography; ECG, electrocardiogram; EEG, electroencephalogram; EMG, electromyography; IgA, immunoglobulin A; IgG, immunoglobulin G; IgM, immunoglobulin M; *MEGF10* , *multiple EGF like domains 10* ; MRI, magnetic resonance imaging; NR, non-reactive; PET, positron emission tomography; RF, rheumatoid factor; SWI, susceptibility weighted imaging; TCD, transcranial doppler ultrasound; VDRL, venereal disease research laboratory test.

Notes: *During the test with a monopolar needle, there was a difference in muscle activation, which suggests central nervous system disease; **echo was limited for the thalamus.

**Figure 1:**
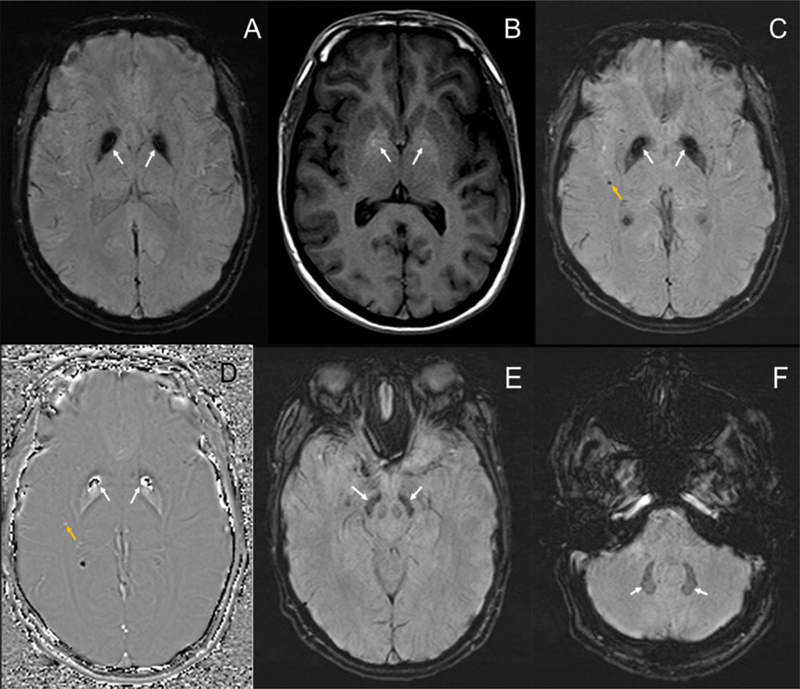
Structural neuroimaging obtained in September 2019 ( **A–D** ) and December 2022 ( **E,F** ). There is significant hypointensity on the susceptibility-weighted imaging (SWI) sequence ( **A** ) and slight hyperintensity on T1 in the globus pallidus ( **B** ), compatible with a mixed anomaly of ferromagnetic deposition and calcifications. Also, on the SWI sequence, there are areas of subinsular hemorrhagic residue to the right and in the left parietal lobe ( **C,D** ), indicated by yellow arrows, and a smaller portion of ferromagnetic deposition in the substantia nigra and dentate nuclei ( **E,F** ).

**Figure 2:**
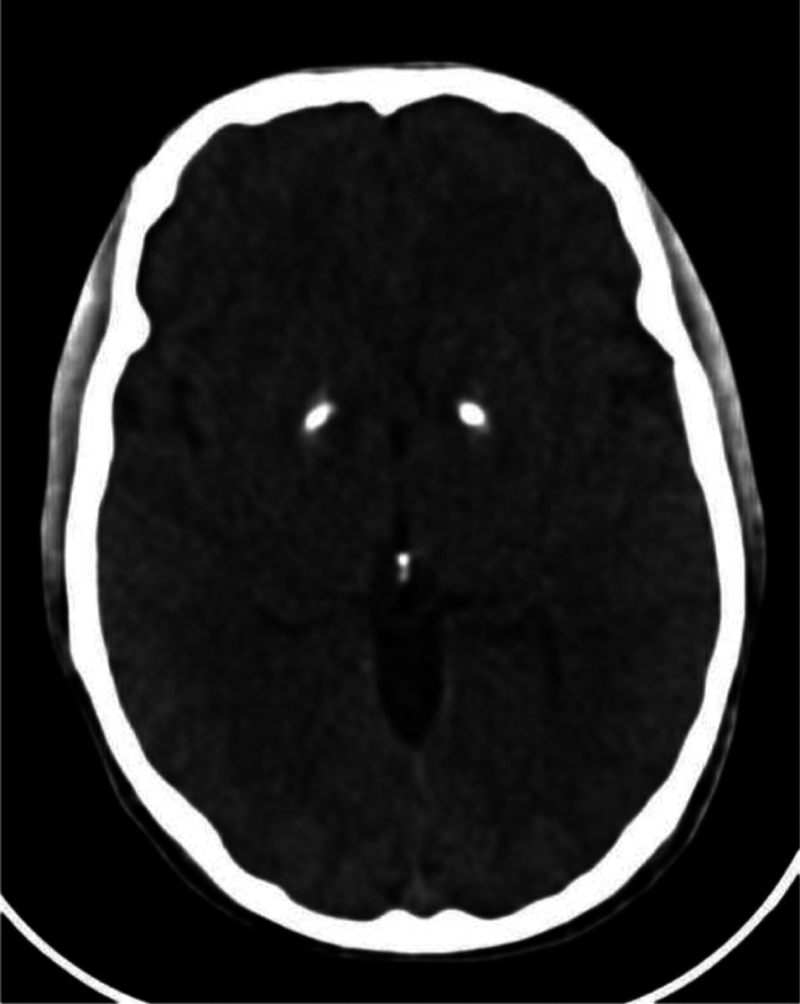
Skull computed tomography (CT) scan (performed in 2016): small and symmetrical calcifications on the basal ganglia.

A brain magnetic resonance imaging (MRI) scan revealed hypointensity on susceptibility-weighted imaging (SWI) and subtle hyperintensity on the T1-weighted sequence in the globus pallidus, suggestive of abnormal iron deposition and calcifications ( Figure 1 ). Furthermore, a cranial computed tomography (CT) scan demonstrated small and symmetrical calcifications on the basal ganglia ( Figure 2 ). These calcifications represent a potential confounder when interpreting the SWI findings. However, their size was smaller than the extent of the SWI hypointensities, which extend beyond the calcified regions. Given the possible overlap of these findings with the diagnosis of neurodegeneration with brain iron accumulation (NBIA), the patient underwent complete exome sequencing and mitochondrial DNA analysis. The exome analysis showed only variants of undetermined significance in the *acyl-CoA dehydrogenase medium chain* ( *ACADM* ) and *multiple EGF like domains 10* ( *MEGF10* ) genes, which do not correlate with our patient's clinical picture. Therefore, the diagnosis of abnormal iron accumulation related to DSRD was our main hypothesis.

Although the patient was already in the sequelae stage, quite compromised, dependent on others for basic daily activities, a case discussion was held with the family. We considered the presence of pleocytosis in previous CSF studies, as well as other nonspecific markers of systemic autoimmunity, as possible indicators of an immune-mediated basis for DSRD in this case. Our patient was prescribed 6 cycles of intravenous immunoglobulin (IVIG) therapy every 4 weeks. Still, no evident responses in cognitive or motor functions were observed, and we decided not to pursue additional immune treatments. Currently, he depends on caregivers for all basic activities of daily living. Still, he has gained a significant quality of life under a multidisciplinary rehabilitation program and is medicated on symptomatic drugs for irritability (sertraline) and dysautonomia (fludrocortisone).

## FROM PRESENTATION TO RESOLUTION: LESSONS LEARNED

### What is DSRD, its etiology, and the main clinical findings?

A rare cause of global neuropsychiatric regression, DSRD is observed in previously-healthy individuals with DS. 1 The clinical manifestations related to DSRD comprise acute or subacute loss of cognitive functions and functionality in previously-healthy individuals with DS, as well as autonomic, motor, and psychiatric symptoms. 1 2 A group of 28 clinical features presented by DSRD patients was proposed in a previous study 2 as a diagnostic aid for clinicians. Presently, however, there are no fully-established diagnostic tools nor criteria for DSRD.

There is considerable debate regarding the etiology of DSRD, including degenerative and autoimmune mechanisms, 3 but it remains uncertain. Among the explanations proposed to support the immunologic hypothesis is the hyperactivation of the Janus kinase-signal transducer and activator of transcription (JAK/STAT) signaling cascades, attributed to interferon receptor genes encoded on chromosome 21. 4 In addition, individuals with DS are much more likely to present with autoimmune diseases, including thyroid autoimmune disease, celiac disease, and skin immunomodulated disorders. 5

The patient herein reported experienced a dramatic neurocognitive decline, with signs and symptoms such as apathy, syncope, sleep disturbances, and extrapyramidal signs, accompanied by neuroimaging findings indicative of abnormal brain iron accumulation. Our experience with diagnosing DSRD involved extensive clinical and laboratory investigations, as well as neuroimaging, to identify differential diagnoses.

### Why is neuroimaging relevant to the diagnosis of DSRD?

The literature on DSRD neuroimaging data indicates that abnormalities in brain MRI scans are among the most common findings in the disorder, in which the most distinctive is the change in signal in the basal nuclei on SWI, which was observed in 74% of 164 patients with the disorder in a retrospective cohort; 5 a small case series 3 also described patients with low signal in T2-weighted images and SWI sequences in the globus pallidus and, to a lesser extent, in the substantia nigra (SN). The underlying mechanism of cerebral alterations in DSRD remains unknown. Besides the likely role of autoimmunity, some studies 3 consider psychological stressors that dysregulate neurotransmitters, such as acetylcholine and serotonin, as potential contributors.

In this context, it is notable that patients with DSRD may exhibit overlapping clinical features with NBIA disorders, particularly motor symptoms such as Parkinsonism. Despite this scenario, iron accumulation appears to be a rare finding in DSRD, 3 while the imaging abnormalities in the cited regions have been hypothesized as immunologically-mediated calcifications linked to interferon signalling. 6 Thus, although these neuroimaging findings may serve as potential markers for diagnostic differentiation in DSRD, their relationship to the underlying etiology and final diagnosis remains unclear. Besides, it is worth noting the limitations of the current study in directly associating the imaging findings herein presented with DSRD-related iron accumulation.

### What other steps can be taken for DSRD investigation?

The diagnosis of DSRD is primarily clinical, based on criteria established by a panel of healthcare professionals experienced in managing neurocognitive regression in individuals with DS. 2 However, since the exclusion of other psychiatric and neurological conditions is fundamental to these criteria, additional diagnostic studies such as blood tests and lumbar puncture may be performed, even though little is known about specific biomarkers in DSRD patients.

In this setting, the possible role of autoimmunity in the disorder can guide clinical investigation, since inflammatory and autoimmune markers, such as positive antinuclear antibodies, low complement component 3 (C3) levels, and elevated ferritin, are often present in patients with DSRD. In addition, previous investigations 7 in patients with the disorder have identified that certain CSF abnormalities indicative of neuroinflammation and increased permeability in the blood-brain barrier, such as pleocytosis, the presence of oligoclonal bands, and an elevated immunoglobulin G (IgG) index. The pleocytosis observed in the patient herein described supported the immune-mediated hypothesis and served as the basis for immunoglobulin therapy.

### How can DSRD be treated?

Currently, the limited number of studies does not enable the establishment of therapeutic guidelines. Nevertheless, the neuroimaging abnormalities and CSF changes described were identified as predictive of response to immunotherapy. 5 8 However, the former is the most statistically significant predictive factor, and it can achieve approximately eight times greater therapeutic response. 9 Immunoglobulin was found to be highly effective in improving symptoms in 88% of the patients in another study. 6 Conversely, in a group of patients with normal neuroimaging, there was a better response rate to benzodiazepines, antipsychotics, and electroconvulsive therapy (ECT). 6

Despite the brain MRI abnormalities, a positive anti-thyroid peroxidase (anti-TPO) test, and mild pleocytosis in one CSF study, our patient did not respond to 6 cycles of intravenous immunoglobulin (IVIG); the therapeutic failure may reflect a neurodegenerative etiology or a chronic-sequelae stage of the disorder. Ultimately, this scenario underscores the heterogeneity of disease presentation and treatment outcomes, emphasizing the need for heightened clinical awareness and further research to clarify the pathophysiology and establish evidence-based management strategies.

## Notes

TEACHING POINTS

1. The etiology of Down syndrome regression disorder (DSRD) remains a debated topic, with probable immune and degenerative components.
2. Neuroimaging findings are important to select therapeutic methods in DSRD.
3. The diagnostic evaluation and management of DSRD require a comprehensive, multidisciplinary approach.
